# Connectivity on Underwater MI-Assisted Acoustic Cooperative MIMO Networks †

**DOI:** 10.3390/s20113317

**Published:** 2020-06-10

**Authors:** Qingyan Ren, Yanjing Sun, Yu Huo, Liang Zhang, Song Li

**Affiliations:** 1 School of Information and Control Engineering, China University of Mining and Technology, Xuzhou 221000, China; qingyanren@cumt.edu.cn (Q.R.); yuhuo@cumt.edu.cn (Y.H.); zhangliang19840425@gmail.com (L.Z.); lisong@cumt.edu.cn (S.L.); 2 Xuzhou Engineering Research Center of Intelligent Industry Safety and Emergency Collaboration, Xuzhou 221000, China; 3 School of Communication and Information Engineering, Xi’an University of Science and Technology, Xi’an 710054, China

**Keywords:** heterogeneous underwater wireless sensor networks, cooperative MIMO, magnetic induction, connectivity

## Abstract

In traditional underwater wireless sensor networks (UWSNs), it is difficult to establish reliable communication links as the acoustic wave experiences severe multipath effect, channel fading, and ambient noise. Recently, with the assistance of magnetic induction (MI) technique, cooperative multi-input-multi-output (MIMO) is utilized in UWSNs to enable the reliable long range underwater communication. Compared with the acoustic-based UWSNs, the UWSNs adopting MI-assisted acoustic cooperative MIMO are referred to as heterogeneous UWSNs, which are able to significantly improve the effective cover space and network throughput. Due to the complex channel characteristics and the heterogeneous architecture, the connectivity of underwater MI-assisted acoustic cooperative MIMO networks is much more complicated than that of acoustic-based UWSNs. In this paper, a mathematical model is proposed to analyze the connectivity of the networks, which considers the effects of channel characteristics, system parameters, and synchronization errors. The lower and upper bounds of the connectivity probability are also derived, which provide guidelines for the design and deployment of underwater MI-assisted acoustic cooperative MIMO networks. Monte Carlo simulations were performed, and the results validate the accuracy of the proposed model.

## 1. Introduction

Underwater wireless sensor networks (UWSNs) have been applied in various oceanography missions such as underwater pollution detection, offshore oil extraction, and surveillance where human operation is impossible [1,2,3,4,5]. Since the main function of UWSNs is to process extracted data and transmit them to remote locations, network connectivity is one of the most fundamental problems in UWSNs [6]. Due to unstable underwater environment, limited availability, and non-rechargeability of sensor nodes, the design of UWSNs would reduce cost, be robust to node failures, reduce communication overhead, and guarantee a high level of network connectivity [7,8,9].

Connectivity is affected by communication range of the sensor node, which ensures that every sensor node is connected to the surface BS directly or through a multi-hop path [10]. Compared with electromagnetic wave (EM), acoustic wave is able to propagate much longer distances in the ocean, which is often used as the media for information exchange in UWSNs. However, the acoustic wave during propagation suffers from multipath effect, channel fading, and ambient noise; as a result, it is a great challenge to establish reliable communication links among different nodes of UWSNs [11,12,13]. Furthermore, the acoustic spectrum becomes more and more scarce with the increasing usage of underwater acoustic systems [14]. The underwater acoustic multi-input-multi-output (MIMO) system may be used to satisfy the long-range and high-throughput requirements [15,16]. However, many wireless devices cannot support multiple antennas due to size, cost, and/or hardware limitations in practice. Hence, cooperative MIMO is adopted, the basic idea of which is to group multiple devices into virtual antenna arrays to emulate MIMO communications. Multiple sensor nodes cooperatively transmit the same signals to a receiver and the receiving power gain is enhanced. Consequently, it can sufficiently increase the range of wireless links without amplifying the transmission power, while saving the energy of each transmitting agent [17]. The virtual antenna array composed of multiple devices in cooperative MIMO relies on wireless communication among the sensor nodes. It requires that all the participating nodes share the same transmitted signals and carrier frequency, and they have accurate synchronous clocks.

**Figure 1:**
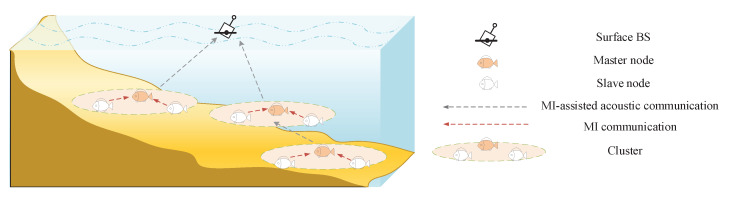
The system architecture of underwater MI-assisted acoustic cooperative MIMO networks.

However, underwater acoustic channels are not proper for the synchronization as the low propagation speed takes long synchronization time and has large synchronization errors. As a popular near-field communication technique, magnetic induction (MI) has great potential in UWSNs because it can provide a constant channel, high data rate, and negligible propagation delay with the underwater propagation speed of $3.33\times 10^{7}$ m/s [18,19,20]. Such high speed signal propagation is able to significantly facilitate the synchronization of distributed nodes, which enables the long distance underwater communication and high throughput. Therefore, heterogeneous wireless underwater sensor networks adopting MI-assisted acoustic cooperative MIMO are proposed [21]. In such networks, the distributed nodes are divided into multiple clusters by region, as shown in Figure 1. Each cluster consists of one master sensor node and multiple slave sensor nodes. The information exchange between different clusters are realized by the cooperative MIMO, while MI communication among intra-cluster nodes guarantee the accurate synchronization. The system architecture and process diagram of underwater MI-assisted acoustic cooperative MIMO networks are introduced in Section 2, and much more details can be also found in [21].

The performance of the underwater MI-assisted acoustic cooperative MIMO networks is obtained in [21], in which the performance gain of underwater MI-assisted acoustic cooperative MIMO networks is validated in terms of synchronization errors, signal-to-noise ratio (SNR), effective communication time, and the upper bound of the throughput in physical layer. In this paper, we focus on the system level performance of underwater MI-assisted acoustic cooperative MIMO networks in terms connectivity and analyze the impact of clock synchronization on connectivity. Because of the complex channel characteristics and the heterogeneous network architecture, the connectivity analysis of MI-assisted acoustic cooperative MIMO networks is much more complicated than that of UWSNs. In particular, the acoustic cooperative MIMO and MI communications are performed in inter-cluster and intra-cluster manners, respectively. Therefore, the underwater acoustic channel and MI channel should both be considered, which increases the complexity of connectivity analysis of the networks. Besides the complex channel characteristics, the heterogeneous architecture of MI-assisted acoustic cooperative MIMO networks also poses a great challenge for connectivity analysis. Compared with the traditional UWSNs [7,22], the inter-cluster and intra-cluster connectivity need to be calculated. Meanwhile, the synchronization error is inevitable in the connectivity analysis of the networks.

Without loss of generality, the networks are supposed to be deployed in the bounded three-dimensional space $R^{3}$. In this underwater MI-assisted acoustic cooperative MIMO networks, the clusters are distributed according to a homogeneous Poisson point process and the nodes in a cluster are distributed according to another homogeneous Poisson point process. When considering the probability that a certain cluster can be directly connected with surface BS, it is not only related to its position in space $R^{3}$, but also related to the maximum transmission range of the cluster, which depends on the number of nodes in the cluster [23]. When considering the multi-hop fashion, some work has been well explored to analyze the connectivity of wireless sensor networks assuming two-dimensional coordinates [24]. We extend it to the three-dimensional scene in this network model and give the upper and lower bounds of connectivity.

The major contributions of this paper can be summarized as follows:

- A mathematical model is developed to analyze the connectivity of underwater MI-assisted acoustic cooperative MIMO networks, which considers the three-dimensional underwater bounded environment. Connectivity probabilities of direct and multi-hop fashions are derived.
- Due to the characteristics of heterogeneous networks, inter-cluster and intra-cluster connectivities need to be calculated, and the influence of synchronization errors on connectivity is also considered. Monte Carlo simulations were carried out, and the results agree well with the analytical model that validate the accuracy of the model.
- Based on the framework, we present numerical and simulation results that quantitatively analyze the effects of various system parameters on the connectivity in this networks, considering the effects of channel characteristics, operating frequency, operating range, and density of nodes. The results provide the guideline for the deployment of underwater MI-assisted acoustic cooperative MIMO networks according to the practical requirement.

The remainder of this paper is organized as follows. In Section 2, a brief introduction to the network architecture of underwater MI-assisted acoustic cooperative MIMO networks is given. Based on the system architecture, the connectivity problem is formulated in Section 3. In Section 4, the transmission ranges of node and cluster are derived. Next, connectivity probabilities of direct and multi-hop fashions are further analyzed in Section 5. In Section 6, the connectivity performance evaluation by Monte Carlo simulations is presented. Finally, the conclusions of this paper are drawn in Section 7.

## 2. System Model

The complex system architecture constitutes one of the major challenges in the connectivity analysis. In this section, we describe the system architecture and channel model, and the synchronization accuracy is analyzed.

### 2.1. System Architecture

The system architecture of underwater MI-assisted acoustic cooperative MIMO networks is illustrated in Figure 1. It consists a surface base station (BS) and a great number of underwater sensor nodes. The surface BS aggregates the data from underwater nodes for environment sensing, which includes water velocity, temperature, salinity, etc. Underwater sensor nodes are managed in the form of clusters with assigned IDs. In each cluster, the sensor selected as the master node collects the monitoring data from slave nodes and coordinatea the intra-cluster synchronization. The acoustic cooperative MIMO is utilized between the surface BS and clusters as well as different clusters for information exchange and command interaction. As the cooperative MIMO technique collects all the intra-cluster nodes power, the long range underwater acoustic communication is enabled, which expands the effective cover space. Compared with traditional UWSNs [1], the cluster of the networks can be considered as an enhanced node due to the adoption of MI-assisted acoustic cooperative MIMO.

**Figure 2:**
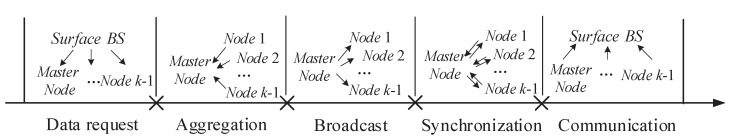
The process diagram of underwater MI-assisted acoustic cooperative MIMO networks.

The process diagram of MI-assisted acoustic cooperative MIMO networks is shown in Figure 2. It includes five phases: *Data request*, *Aggregation*, *Broadcast*, *Synchronization*, and *Communication*. During the *Data request* period, the BS sends the data request to clusters. As the cluster receives the request, the monitoring data are transmitted from slave nodes to the master node by MI communication during the *Aggregation* period. The master node aggregates the monitoring data from different slave nodes and then broadcasts them to all the slave nodes during the *Broadcast* period. As a result, all the intra-cluster slave nodes share the same monitoring data. Timing-sync Protocol for Sensor Networks (TPSN) is adopted to adjust the clocks of slave nodes to that of the master node, which is operated in the *Synchronization* period [25]. After the *Synchronization* period, all the nodes within a cluster have the same monitoring data as well as the same clock. Finally, the monitoring information converged to the surface BS via the direct link or the multi-hop fashion in the *Communication* period.

### 2.2. Channel Characteristics

There are two types of channel in the underwater MI-assisted acoustic cooperative MIMO networks: the underwater acoustic channel and the magnetic induction channel.

In underwater MI-assisted acoustic cooperative MIMO networks, underwater acoustic communication is adopted for communication between clusters in terms of cooperative MIMO. In this paper, we consider Thorp’s underwater acoustic channel model [26], which is based on empirical data. Thorp’s model provides the attenuation for the underwater acoustic channel, which is influenced by the frequency and the communication distance between the sender node and the receiver node. The transmission power model is formulated as [26]

(1) $$P_{r,ac}=\frac{P_{t}}{A(d,f)},$$

where $P_{r,ac}$ is received power, $P_{t}$ is the transmitted power, and A(d,f) is the channel attenuation depending on the distance *d* and the frequency *f* (kHz)

(2) $$A\left(d,f\right)=A_{0}d^{\theta }a{\left(f\right)}^{d/10^{3}},$$

where $A_{0}$ is the normalizing constant and θ is the spreading factor. The spreading factor has a value between 1 and 2 depending on the depth. a(f) is the absorption coefficient and is expressed in decibels per kilometer

(3) $$10\log a\left(f\right)=0.11\frac{f^{2}}{1+f^{2}}+44\frac{f^{2}}{4100+f^{2}}+2.75\times 10^{-4}f^{2}+0.003.$$

In underwater MI-assisted acoustic cooperative MIMO networks, MI is adopted for information aggregation and synchronization within a cluster. As a near-filed communication technique, the transmission power model is formulated as [27]

(4) $$P_{r,MI}=\frac{P_{t}}{r^{6}}\cdot \rho ,$$

where $P_{r,MI}$ is received power, $P_{t}$ is the transmitted power, and *r* is the distance of the master node and one slave node; $\rho =Q_{T}Q_{R}\eta_{T}\eta_{R}r_{T}^{3}r_{R}^{3}\pi^{2}G$ where $Q_{T}=\omega L_{T}/R_{LT}\;$ and $Q_{R}=\omega L_{R}/R_{LR}$ are quality factors of transmitter and receiver, respectively; $L_{T}$ and $L_{R}$ are the inductances of the transmitting and receiving coils; $R_{LT}$ and $R_{LR}$ are the transmitting and receiving coil resistances; $\eta_{T}=R_{S}/\left(R_{S}+R_{LT}\right)\;$ and $\eta_{R}=R_{L}/\left(R_{L}+R_{LR}\right)\;$ are efficiencies of the transmitter and receiver coils; $R_{S}$ and $R_{L}$ are source and load resistances; $r_{T}$ and $r_{R}$ are the radius of the transmitting and the receiving coil; ω is resonant frequency; $G=e^{-r/\delta \;}$ is a parameter related to the channel state; and δ is conductivity. For sea water, fresh water, and dry oil, δ is δ=4.5 S/m, δ=0.07 S/m, and δ=0.0048 S/m, respectively.

Highly conductive sea water induces significant eddy current that incurs very high path loss. Therefore, in seawater, the path loss of MI communication also needs to take into account the influence of medium loss [28]. The attenuation coefficient is

(5) $$\alpha =\sqrt{\pi f\mu_{0}\delta },$$

where $\mu_{0}$ is vacuum permeability.

Therefore, the path loss caused by the influence of seawater medium $PL_{\alpha }$ (dB) is

(6) $$PL_{\alpha }=20\log e^{\alpha r}\approx 8.69\alpha r.$$

Finally, the path loss of MI communication in seawater is

(7) $$\begin{array}{c} PL_{\mathrm{sea}}=PL_{1}+PL_{\alpha }\\ \;\;\;\;\;\;=-10\log\frac{P_{r}}{P_{t}}+8.69\alpha r. \end{array}$$

### 2.3. Synchronization Accuracy Analysis

For the TPSN synchronization algorithm [25], the clock drift of two nodes is assumed to be unchanged in this small span of time. As shown in Figure 2, *k* − 1 slave nodes adjust their clocks to that of the master node in turn. The node *z* is taken as an example to illustrate the TPSN synchronization process. The node *z* sends *synchronization-pulse* packet to the master node at $T_{1}$, and this package contains time $T_{1}$. The master node receives the packet at $T_{2}$, where $T_{2}$ is equal to $T_{1}+\Delta_{1}+\Delta_{2}$. Here, $\Delta_{1}$ and $\Delta_{2}$ represent the clock drift between the two nodes and propagation delay, respectively. The master node returns an *acknowledgement* packet to the node *z* at $T_{3}$, which contains the values of $T_{1}$, $T_{2}$, and $T_{3}$, as well as beacon signal $f_{s}$ generated by the master node. The node *z* receives the packet at $T_{4}$. The clock drift and propagation delay of the node *z* can be calculated as

(8) $$\Delta_{1}=\frac{\left(T_{2}-T_{1}\right)-\left(T_{4}-T_{3}\right)}{2},$$

(9) $$\Delta_{2}=\frac{\left(T_{2}-T_{1}\right)+\left(T_{4}-T_{3}\right)}{2}.$$

As the drift is indicated in Equations (8) and (9), the node *z* is able to correct its clock aligned with that of the master node.

However, the node’s clock is driven by crystal oscillation. Doe to the existence of impure crystals, environmental changes, and component aging, frequency and synchronization errors are unavoidable. First, the frequency error of the synchronization is analyzed herein [21]. The average relative clock drift in ΔT of the node *z* to the master node can be expressed as

(10) $$\bar{a_{z}}=\frac{\int_{\Delta T}a_{z}\left(t\right)dt}{\Delta T}=\frac{\int_{\Delta T}\frac{f_{c,z}\left(t\right)}{f_{c,0}\left(t\right)}dt}{\Delta T},$$

where $f_{c,z}\left(t\right)$ is the oscillator frequency of the node *z* and $f_{c,0}\left(t\right)$ is the oscillator frequency of the master node.

For node *z*, its working frequency $f_{s,z}\left(t\right)$ is proportional to its crystal oscillator frequency, thus

(11) $$f_{s,z}\left(t\right)=K\cdot f_{c,z}\left(t\right)\;\;\forall z=1,2,\ldots N,$$

where *K* is the amplification factor of the frequency multiplier.

After receiving the *acknowledgement packet* at the node *z*, the frequency is estimated according to the local oscillator of the node

(12) $${\hat{f}}_{s,z}=\bar{a_{z}}f_{s}+\varepsilon_{s,z},$$

where $f_{s}$ is the working frequency of the master node and $\varepsilon_{s,z}$ is the error of the frequency estimation.

The frequency offset at the node *z* for the frequency synchronization can be determined as

(13) $$\Delta f_{c,z}=\frac{{\hat{f}}_{s,z}-f_{s}}{K}=\left(\bar{a_{z}}-1\right)f_{s}+\frac{\varepsilon_{s,z}}{K}.$$

According to Equation (13), the optimal frequency offset is $\left(\bar{a_{z}}-1\right)f_{s}$. Due to the estimated error $\varepsilon_{s,z}$, the frequency cannot be perfectly synchronized and the error can be defined as

(14) $$\varepsilon_{z,f}=\frac{\varepsilon_{s,z}}{K}.$$

For the node *z*, the duration of the time slot is $\Delta t_{z}$, which mainly includes the sending time and transmission time of *synchronization-pulse* packet and *acknowledgement* packet

(15) $$\Delta t_{z}=\frac{L_{z,0}+L_{0,z}}{B}+\frac{2r_{0,z}}{v},$$

where $L_{z,0}$ and $L_{0,z}$ are the size of *synchronization-pulse* packet and *acknowledgement* packet, *B* is the bandwidth, $r_{0,z}$ is the distance between master node and node *z*, and *v* is the propagation speed of signal.

Due to the frequency error of crystal oscillator at each node, the total drifting time of each node will gradually increase along with time. For the node *z*, the time synchronization error to the beginning of beamforming can be calculated by

(16) $$\varepsilon_{z,t}=\varepsilon_{z,f}\left(\frac{L_{0,z}}{B}+\frac{r_{0,z}}{c}+\sum_{i=z+1}^{k-1}\Delta t_{i}\right).$$

## 3. Problem Formulation

Based on the system architecture and operation mechanism of underwater MI-assisted acoustic cooperative MIMO networks given in Section 2, the problem of connectivity analysis is formulated in this section.

Without loss of generality, the network is supposed to be deployed in a bounded 3D space $R^{3}$. In this area, the clusters $\left\{N_{j},j=1,2,\cdots \right\}$ are regarded as independent units, which are distributed according to a homogeneous Poisson point process with the density of $\rho_{c}$. The nodes $\left\{N_{i},i=1,2,\cdots \right\}$ in one certain cluster are subject to another homogeneous Poisson distribution with the density of $\rho_{n}$.

In MI-assisted acoustic cooperative MIMO networks, a cluster with *k* nodes and the surface BS can successfully establish a connected link if and only if the following two conditions are satisfied: (1) k−1 slave nodes can connect topologically with the master node; and (2) the cluster with *k* nodes participating in cooperative MIMO and the surface BS can connect topologically via a direct link or a multi-hop fashion. Specifically, the definitions of node connected and cluster connected are provided as follow [24]:

**Definition** **1.** *When there is a master node within the maximum transmission range of the slave node, it is considered that it can connect to the master node, and the slave node is defined as connected.*

**Definition** **2.** *When the surface BS is within the maximum transmission range of the cluster, it is considered that it can connect to the surface BS directly, and the cluster is defined as connected.*

**Definition** **3.** *When there are relays that can connect topologically with each other until the surface BS, it is considered that it can connect to the surface BS in multi-hop fashion, and the cluster is defined as connected.*

From the above definitions, the connectivity of the *j*th cluster in the networks can be defined as

(17) $$P_{C,j}=\sum_{k=0}^{\infty }P_{N,k}\cdot P_{C,k,j},$$

where $P_{C,k,j}^{}$ is probability that the *j*th cluster including *k* nodes connects to the surface BS, denoted as Equation (18), and $P_{N,k}^{}$ is the probability that there are *k* nodes participating in the cooperative MIMO in the *j*th cluster, indicated as Equation (19). Specifically,

(18) $$P_{C,k,j}^{}=\int_{R^{3}}\frac{1}{V_{R^{3}}}P\left(x_{j}↔\mathrm{surfaceBS}\right)dx_{j},$$

where $x_{j}$ is the position of the *j*th cluster, and $P(x_{j}↔\mathrm{surfaceBS})$ is the probability that the *j*th cluster at $x_{j}$ is connected to the surface BS, which depends on the distribution and maximum transmission range of the cluster. As the maximum transmission range of the cluster depends on the number of nodes participating in the cooperative MIMO, the maximum transmission range of the cluster with *k* nodes is recorded as $R_{ac,k}$.

According to Definition 1, we have

(19) $$P_{N,k}=P\left(\mathrm{All}\;k\;\mathrm{nodes}\;\mathrm{are}\;\mathrm{connected}\right)\cdot P\left(\mathrm{There}\;\mathrm{are}\;k\;\mathrm{nodes}\right)=\frac{{\left(\rho_{n}S\right)}^{k}}{k!}\cdot e^{-\rho_{n}S},$$

where $S=\pi R_{\mathrm{MI}}^{2}$ and $R_{\mathrm{MI}}^{}$ is the maximum transmission range of the node. Substituting Equations (18) and (19) into Equation (17), we have

(20) $$P_{C,j}=\sum_{k=0}^{\infty }\frac{{\left(\rho_{n}\pi R_{\mathrm{MI}}^{2}\right)}^{k}}{k!}e^{-\rho_{n}\pi R_{\mathrm{MI}}^{2}}\cdot \int_{R^{3}}\frac{1}{V_{R^{3}}}P\left(x_{j}↔\mathrm{surfaceBS}\right)dx_{j}.$$

From Equation (20), to derive the connectivity probability $P_{C,j}$, the following terms are requested: the maximum transmission range of the node $R_{\mathrm{MI}}$, the maximum transmission range of the cluster $R_{ac,k}$, and the probability that the *j*th cluster at $x_{j}$ connects to the surface BS $P(x_{j}↔\mathrm{surfaceBS})$. These are analyzed in the following sections.

## 4. Transmission Range

### 4.1. Inter-Cluster Acoustic Transmission Range

It is assumed that there are *k* nodes distributed in the *j*th cluster. Let $\tau_{i}$ denote the time delay of the *i*th sensor, where i=1,2,⋯,k. The signal from the *k* nodes arrives at the surface BS can be written as

(21) $$y\left(t\right)=\sum_{i=1}^{k}H_{i}h_{i}e^{j2\pi f(t-\tau_{i})}+n\left(t\right),$$

where $H_{i}$ is the amplitude of the *i*th transmitted signal, $h_{i}=1/\sqrt{A(d_{i},f)}$ is the underwater acoustic channel envelope [26], *f* is the signal frequency, and n(t) is the noise. Similar to the assumption in [21,29], as *k* nodes share the same transmitted signal, the amplitude $H_{i}$ can be recorded as *H*. Considering the surface BS deployed in the far-field, all the channel envelops are the same.

Therefore, the signal at the surface BS can be rewritten as

(22) $$y\left(t\right)=\sum_{i=1}^{k}Hhe^{j2\pi f(t-\tau_{i})}+n\left(t\right).$$

We consider the perfect CSI information, the channel envelope $h_{i}$ and the delay $\tau_{i}$ are known. From Equation (22), the received signals by surface BS cannot be enhanced as the phase difference caused by the time delay. To maintain the reliable communication, the clusters should be close to the surface BS that leads to a limited effective cover space. To tackle this problem, pre-compensation phases are added to the nodes to eliminate the time delays, and this technique is referred as cooperative MIMO. After the phase compensation, the received signal at the surface BS can be expressed as

(23) $$y\left(t\right)=\sum_{i=1}^{k}Hhe^{j2\pi ft}w_{i}e^{-j2\pi f\tau_{{}_{i}}}+n\left(t\right),$$

where $w_{i}\in C$ is the pre-compensation phase and λ is the wave length. Define $w={\left[w_{1}\cdots \omega_{k}\right]}^{T}$, $a_{\phi }={\left[\;e^{-j2\pi f\tau_{{}_{1}}}\;\ldots \;e^{-j2\pi f\tau_{{}_{i}}}\right]}^{T}$, and $\sigma^{2}=E\left\{{\left|n(t)\right|}^{2}\right\}$. The received signal y(t) can be expressed in a compact form [29]:

(24) $$y\left(t\right)=Hhe^{j2\pi ft}w^{T}a_{\phi }+n\left(t\right).$$

Then, the SNR of received signal at the surface BS can be calculated as

(25) $$SNR_{ac}=\frac{{\left|Hhe^{j2\pi ft}w^{T}a_{\phi }\right|}^{2}}{\sigma^{2}}.$$

The optimal compensation vector w can be obtained by solving the following optimization problem:

(26) $$\begin{array}{c} \max_{w}\;\;\;\frac{{\left|Hhe^{j2\pi ft}w^{T}a_{\phi }\right|}^{2}}{\sigma^{2}}\\ s.t.\;{\left\|w\right\|}^{2}\leq 1. \end{array}$$

The optimization problem in Equation (26) can be solved by the Lagrangian multiplier method, and the optimal compensation vector is given as below [29]

(27) $$w^{*}=\frac{1}{\sqrt{k}}{a_{\phi }}^{H}.$$

The above analysis does not consider the synchronization error. Due to the existence of the impure crystals, component aging, and time drifting, frequency and time estimation errors are unavoidable. Taking account of the frequency and time estimation errors, the signals received at the surface BS can be expressed as

(28) $$y\left(t\right)=h\circ w^{H}a_{\phi }+n\left(t\right),$$

where $h=[Hhe^{j2\pi \left(f+\varepsilon_{1,f}\right)\left(t+\varepsilon_{1,t}\right)}\cdots Hhe^{j2\pi \left(f+\varepsilon_{k,f}\right)\left(t+\varepsilon_{k,t}\right)}]$, ∘ is Hadamard product. $\varepsilon_{i,f}$ is frequency estimation error of the *i*th node and $\varepsilon_{i,t}$ is the time estimation error of the *i*th node, which are explained in Section 2.

Therefore, the SNR of signals received at the surface BS is given as [21]

(29) $$SNR_{ac,\varepsilon (t)}=\frac{{\left|\sum_{i=1}^{k}Hhe^{j2\pi \left(f+\varepsilon_{i,f}\right)\left(t+\varepsilon_{i,t}\right)}\right|}^{2}}{\sigma^{2}}.$$

Based on Equation (29) the maximum transmission range of a cluster is determined as

(30) $$R_{ac}=\max\left\{d:SNR_{ac,\varepsilon (t)}>SNR_{ac,th}\right\},$$

where $SNR_{ac,th}$ indicates the minimum SNR that the surface BS is able to receive and recover the transmitted signal from the cluster.

### 4.2. Intra-Cluster MI Transmission Range

In underwater MI-assisted acoustic cooperative MIMO networks, within a cluster MI is adopted for information aggregation and synchronization. To obtain the intra-cluster transmission range, the SNR of MI is needed. From the transmission power model in Equation (4) in Section 2, the SNR of the signal received by the master node can be expressed as

(31) $$SNR_{MI,\varepsilon (t)}=\frac{P_{t}\rho }{r^{6}\sigma_{MI}^{2}}.$$

Furthermore, the maximum transmission range of intra-cluster communication can be provided as

(32) $$R_{MI}=\max\left\{r:SNR_{MI,\varepsilon (t)}>SNR_{MI,th}\right\},$$

where $SNR_{MI,th}$ indicates the minimum SNR that the master node is able to receive and recover the transmitted signal from slave nodes.

## 5. Connectivity Analysis

From the problem formulation in Section 3, the probability $P_{C,k,j}$ participates in the connectivity analysis. This section provides the derivations of $P_{C,k,j}$ in direct and multi-hop fashions. As the analysis form of $P_{C,k,j}$ in multi-hop is very complicated, the lower and upper bounds are developed, which can provide the guidelines for the networks design in practice.

### 5.1. Directly Connected

**Figure 3:**
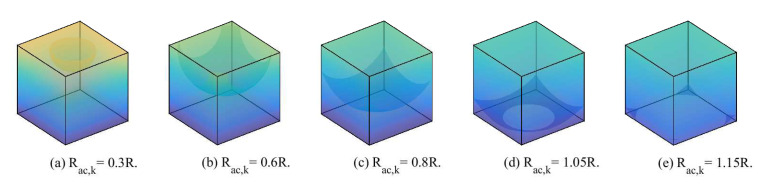
The geometries of underwater MI-assisted acoustic cooperative MIMO networks when directly connected.

Without loss of generality, it is supposed that the $R^{3}$ is a $R^{3}$ cube. There is a surface BS and *m* clusters (m=1,2,⋯) distributed in the $R^{3}$. According to the homogeneous distribution of the clusters, $P_{C,k,j}$ is the probability that the *j*th cluster falls into the hemisphere with surface BS as the center of the sphere and the radius of $R_{ac,k}$. We define the area $Z^{3}$ where clusters can establish a direct link with surface BS. Therefore, $P_{C,k,j}^{}$ can be derived as

(33) $$P_{C,k,j}=\frac{V_{Z^{3}}}{V_{R^{3}}},$$

where $V_{R^{3}}=R^{3}$. However, when considering the boundary effect, the transmission range $R_{ac,k}$ gets larger with the increase of the number of intra-cluster nodes. Based on the values of $R_{ac,k}^{}$ and *R*, $V_{Z^{3}}$ can be calculated as:

**Case 1**: When $0\leq R_{ac,k}<\frac{R}{2}$, $Z^{3}$ is a hemisphere, as shown in Figure 3a. We have

(34) $$V_{Z^{3}}=\frac{2\pi R_{ac,k}^{3}}{3}.$$

**Case 2**: When $\frac{R}{2}\leq R_{ac,k}<\frac{\sqrt{2}R}{2}$, the hemisphere partially extends beyond the sides of the cube to form four hemispheric crowns, as shown in Figure 3b. We have

(35) $$V_{Z^{3}}=\frac{2\pi R_{ac,k}^{3}}{3}-\frac{2\cdot \pi {\left(\frac{R}{2}-R_{ac,k}\right)}^{2}\left(2R_{ac,k}+\frac{R}{2}\right)}{3}.$$

**Case 3**: When $\frac{\sqrt{2}R}{2}\leq R_{ac,k}<R$, the hemisphere extends beyond the sides of the cube and does not reach the bottom of the cube, as shown in Figure 3c. $V_{Z^{3}}$ is calculated by calculus. We have

(36) $$V_{Z^{3}}=4\int_{0}^{\frac{R}{2}}\int_{0}^{\frac{R}{2}}\sqrt{R_{ac,k}^{2}-x^{2}-y^{2}}dxdy.$$

**Case 4**: When $R\leq R_{ac,k}<\frac{\sqrt{5}R}{2}$, the hemisphere extends beyond the sides of the cube and extends beyond the bottom of the cube to form a spherical crown, as shown in Figure 3d. We have

(37) $$V_{Z^{3}}=4\int_{0}^{\frac{R}{2}}\int_{0}^{\frac{R}{2}}\sqrt{R_{ac,k}^{2}-x^{2}-y^{2}}dxdy-\frac{\pi }{3}{\left(R_{ac,k}-R\right)}^{2}\left(2R_{ac,k}+R\right).$$

**Case 5**: When $\frac{\sqrt{5}R}{2}\leq R_{ac,k}<\frac{\sqrt{6}R}{2}$, $R_{ac,k}$ continues to increase, the hemisphere does not completely contain the entire cube, as shown in Figure 3e. We have

(38) $$V_{Z^{3}}=R^{3}-4\int_{\sqrt{R_{ac,k}^{2}-\frac{5}{4}R^{2}}}^{\frac{R}{2}}\int_{\sqrt{R_{ac,k}^{2}-R^{2}-y^{2}}}^{\frac{R}{2}}\left(R-\sqrt{R_{ac,k}^{2}-x^{2}-y^{2}}\right)dxdy.$$

**Case 6**: When $R_{ac,k}\geq \frac{\sqrt{6}R}{2}$, the cluster at any point in $R^{3}$ can be connected to BS, and then $V_{Z^{3}}=R^{3}$.

Based on the above analysis, $P_{C,k,j}^{}$ can be calculated as Equation (39).

(39) PC,k,j={23πRac,k323πRac,k3R3R30≤Rac,k<R223πRac,k3−2·π(R2−Rac,k)2(2Rac,k+R2)323πRac,k3−π(2Rac,k+R2)(R−2Rac,k)6R3R3R2≤Rac,k<2R24∫0R2∫0R2Rac,k2−x2−y2dxdy4∫0R2∫0R2Rac,k2−x2−y2dxdyR3R32R2≤Rac,k<R4∫0R2∫0R2Rac,k2−x2−y2dxdy−π3(Rac,k−R)2(2Rac,k+R)4∫0R2∫0R2Rac,k2−x2−y2dxdy−π3(Rac,k−R)2(2Rac,k+R)R3R3R≤Rac,k<5R2R3−4∫Rac,k2−54R2R2∫Rac,k2−R2−y2R2R−Rac,k2−x2−y2dxdyR3−4∫Rac,k2−54R2R2∫Rac,k2−R2−y2R2R−Rac,k2−x2−y2dxdyR3R35R2≤Rac,k<6R21Rac,k≥6R2.

### 5.2. Lower Bound of Connectivity Probability in Multi-Hop Fashion

Assuming there is a connected link with a relay cluster *B* between the cluster *A* and surface BS, there are *a* nodes in cluster *A* and *b* nodes in relay cluster *B*. Then, the connectivity probability can be calculated as

(40) $$P=\sum_{a=0}^{\infty }\sum_{b=0}^{\infty }P_{a}P_{b}P\left(A↔B\right)P\left(B↔\mathrm{surfaceBS}\right),$$

where $P_{a}$ is the probability that the cluster *A* has *a* nodes, $P_{b}$ is the probability that the relay *B* has *b* nodes, P(A↔B) is the probability that the cluster *A* is connected with relay *B*, and P(B↔surfaceBS) is the probability that the relay *B* is connected with surface BS. Furthermore,

(41) $$\begin{array}{cc} P & =\sum_{a=0}^{\infty }\sum_{b=0}^{\infty }P_{a}P_{b}P\left(A↔B\right)P\left(B↔\mathrm{surfaceBS}\right)\\ \; & \geq \sum_{a=0}^{\infty }P_{a}^{2}P\left(A↔B\right)P\left(B↔\mathrm{surfaceBS}\right). \end{array}$$

From the above analysis, the lower bound of the connectivity probability can be obtained by assuming that all clusters have the same number of nodes, which means that the maximum communication range of all clusters is the same.Therefore, we can obtain

(42) $$P_{C,j}=\sum_{k=0}^{\infty }P_{N,k}\cdot P_{C,k,j}^{{}^{\prime }},$$

where $P_{C,k,j}^{{}^{\prime }}$ is the connectivity probability that the cluster with *k* nodes is connected with surface BS in multi-hop fashion, and it is worth noting that the number of nodes in the cluster as a relay is also *k*.

**Figure 4:**
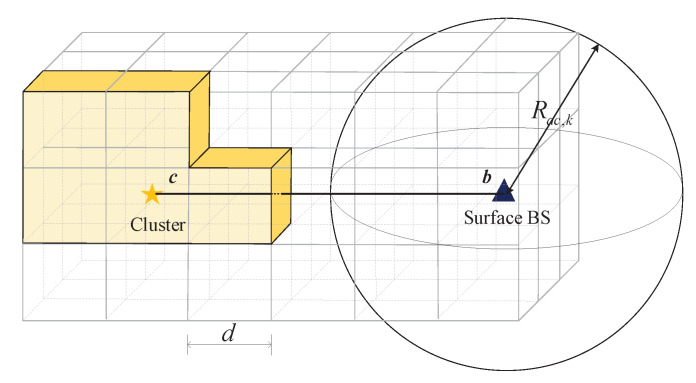
The geometry of underwater MI-assisted acoustic cooperative MIMO networks in multi-hop fashion.

To derive the lower bound of connectivity probability of *j*th cluster with *k* nodes $P_{C,k,j}^{{}^{\prime }}$, we first map the networks on a discrete cube, as shown in Figure 4 The straight line *cb* connecting the *j*th cluster and the surface BS is set as the horizontal line. The *j*th cluster is located at the center of the cube and sides of the cube are parallel to *cb*. The length of each side is $d=\frac{\sqrt{6}}{6}R_{ac,k}$, which means that clusters in two adjacent cubes can be connected with each other. The clusters are distributed according to a homogeneous Poisson point process; as a result, they can be randomly divided into different cubes.

If the *j*th cluster can connect with the surface BS through multi-hop, there could be one or more paths that are distinguished by different relay choices. The set of these paths is denoted as $P_{c}=\left\{P_{c}^{1},P_{c}^{2},\cdots \right\}$, where $P_{c}^{1},P_{c}^{2},\cdots$ denote all possible paths. When the cluster density is high, we use the maximum probability that a certain path exists as the lower bound of $P_{c}$, i.e., $\max_{i}\left\{P\left(P_{c}^{i}\right)\right\}$. When the cluster density is low, we calculate the lower bound of the probability that there is at least one open path, i.e., 1 − $P(|P_{c}|=0)$. The lower bound is $P_{c}$, where $\left|P_{c}\right|$ is the number of the existing paths and $|P_{c}|=0$ means that there is no paths between the *j*th cluster and surface BS.

To get the valid lower bound of connectivity probability $P(P_{c})$, the larger of the two bounds is selected as the lower bound of $P(P_{c})$. Therefore,

(43) $$P\left(x_{j}↔\mathrm{surfaceBS}\right)\geq \max\left\{\max_{i}\left\{P\left(P_{c}^{i}\right)\right\},1-P\left(|P_{c}|=0\right)\right\}.$$

For the different paths that the *j*th cluster can connect with surface BS, the shorter path can yield the higher connectivity probability. Therefore, the shortest path of the relay nodes that exist in the cubes for which a straight line cb passes can yield the maximum existing probability. The number of cubes *W* can be derived as

(44) $$W=\{\begin{array}{cc} \lceil \frac{D-R_{ac,k}}{d}\rceil +1 & D\geq R_{ac,k}\\ 0 & D<R_{ac,k} \end{array},$$

where *D* is the distance between the *j*th cluster and the surface BS and $\left\lceil a\right\rceil$ means rounding *a* to the nearest integer no less than *a*.

When the cluster density is high, the lower bound of connectivity probability is

(45) $$\begin{array}{c} \underset{i}{\max\;}\{P(P_{c}^{i})\}\\ =P^{W}\left(\mathrm{There}\;\mathrm{exists}\;\mathrm{at}\;\mathrm{least}\;\mathrm{one}\;\mathrm{cluster}\;\mathrm{in}\;a\;\mathrm{cube}\right)\\ ={\left(1-q\right)}^{W}, \end{array}$$

where $q=e^{-\rho_{c}d^{3}}$ is the probability that there is no cluster in a cube.

When the cluster density is low, we need to calculate the probability $P(|P_{c}|=0)$. When the *j*th cluster cannot connect with the surface BS, there must be a hull *C* that contains the *j*th cluster and excludes the surface BS, as shown in Figure 4. The cubes inside the hull contain at least one cluster, while those cubes outside the hull have no cluster. Hence, $P(|P_{c}|=0)$ can be evaluated by counting the number of hulls and the hulls must be passed by the straight line cb.

Firstly, the number of faces of a hull is denoted as *n*, the number of hulls that begin at a face and has *n* faces is denoted as σ(n). Obviously, σ(n) is less than the number of self-avoiding walks beginning at a face, i.e., $\sigma \left(n\right)\leq 8\cdot 7^{n-1}$. The total number of such closed hulls is denoted as CN

(46) $$CN\leq \sum_{n=6}^{\infty }\sigma (n-1)+\sum_{n=10}^{\infty }\sigma (n-1)+\cdots +\sum_{n=4W+2}^{\infty }\sigma (n-1).$$

There are relays on one side of the hull and no relays on the other side. Hence, the probability that the *j*th cluster cannot connect with the surface BS $P(|P_{c}|=0)$ is

(47) $$\begin{array}{cc} P(|P_{o}|=0)\; & \leq \sum_{i=1}^{W}\sum_{n=4i+2}^{\infty }8\cdot 7^{n-1}q^{n}\\ \; & =\{\begin{array}{cc} \frac{8\cdot 7^{5}\cdot q^{6}}{1-7q}\cdot \frac{1-{\left(7q\right)}^{4W}}{1-{\left(7q\right)}^{4}} & if\;q<\frac{1}{7}\\ {\left(1-q\right)}^{W} & if\;q\geq \frac{1}{7} \end{array}. \end{array}$$

Substituting Equations (45) and (47) into Equation (43), we have

(48) $$\begin{array}{c} P(x_{j}↔\mathrm{surfaceBS})\\ \geq \{\begin{array}{cc} \max\{{\left(1-q\right)}^{W},1-\frac{8\cdot 7^{5}}{1-7q}\cdot \frac{1-{\left(7q\right)}^{4W}}{1-{\left(7q\right)}^{4}}\} & if\;q<\frac{1}{7}\\ {\left(1-q\right)}^{W} & if\;q\geq \frac{1}{7} \end{array}. \end{array}$$

### 5.3. Upper Bound of Connectivity Probability in Multi-Hop Fashion

To get the upper bound of connectivity probability, we consider the probability in terms of hops. Therefore, the connectivity probability of the *j*th cluster is

(49) $$P=P_{0}+P_{1}+P_{2}+\cdots ,$$

where $P_{0}$ is probability that the *j*th cluster is connected with surface BS directly, $P_{1}$ is probability that the *j*th cluster is connected with surface BS through one relay, and $P_{2}$ is probability that the *j*th cluster is connected with surface BS through two relays. From Equation (49), we can derive that the more paths there are, the greater is the probability of connectivity. The connectivity probability increases with the number of interconnected nodes until there are no isolated nodes. The probability that there are no isolated clusters is the upper bound for the connectivity probability:

(50) $$\begin{array}{c} P_{C,j}\leq P\left(\mathrm{No}\;\mathrm{isolated}\;\mathrm{cluster}\right)\\ \;\;=\sum_{m=0}^{\infty }P(\mathrm{All}\;m\;\mathrm{cluster}\;\mathrm{are}\;\mathrm{not}\;\mathrm{isolated})\\ \;\;\;\;\;\cdot P(\mathrm{There}\;\mathrm{are}\;m\;\mathrm{cluster})\\ \;\;=\sum_{m=0}^{\infty }P^{m}(\mathrm{The}\;\mathrm{cluster}\;\mathrm{is}\;\mathrm{not}\;\mathrm{isolated})\\ \;\;\;\;\;\cdot P(\mathrm{There}\;\mathrm{are}\;m\;\mathrm{cluster}). \end{array}$$

These clusters are distributed according to a homogeneous Poisson point process with density $\rho_{c}$,

(51) $$P_{C,j}\leq \exp\{-\rho_{c}V_{R^{3}}P(\mathrm{The}\;\mathrm{cluster}\;\mathrm{is}\;\mathrm{isolated})\},$$

where the probability of the cluster is isolated is

(52) $$P\left(\mathrm{The}\;\mathrm{cluster}\;\mathrm{is}\;\mathrm{isolated}\right)=\exp\{-\rho_{c}\cdot \frac{4}{3}\pi R_{ac,k}^{3}\}.$$

Substituting Equation (52) into Equation (51), we have

(53) $$P_{C,j}\leq \exp\{-\rho_{c}V_{R^{3}}\cdot \exp\{-\rho_{c}\cdot \frac{4}{3}\pi R_{ac,k}^{3}\}\}.$$

## 6. Simulation Results

To evaluate the accuracy of developed mathematical models, some simulations were carried out. The simulation conditions were set as follows. The $R^{3}$ was a 20 km × 20 km × 20 km cube. The clusters were deployed according to a homogeneous Poisson point process with the density of $\rho_{c}$, while the intra-cluster nodes were distributed according to another homogeneous Poisson point process with the density of $\rho_{n}$. The transmitting power of each node was 10 dBm, and the underwater noise level was −83 dBm. The MI communication was operated at the frequency of 10 MHz with the bandwidth of 20 kHz. The underwater acoustic communication was performed at the frequency of 10 kHz with the bandwidth of 10 kHz. The frequency of crystal oscillator used for nodes’ clock synchronization was 100 kHz. The packet lengths of $L_{0,z}$ and $L_{z,0}$ were set to 100 bits. The propagation speeds of MI and sound were $3.33\times 10^{7}$ m/s and 1500 m/s, respectively.

### 6.1. Transmission Range

**Figure 5:**
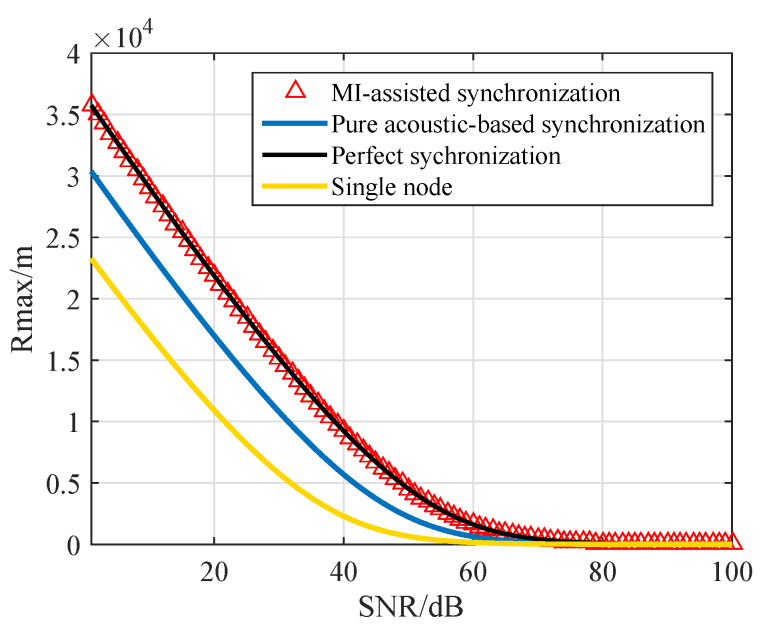
The inter-cluster maximum transmission range.

The maximum transmission ranges of the cluster are plotted in Figure 5. With the increase of SNR threshold, the maximum transmission range decreases subsequently, which is consistent with the analysis. At the same time, connectivity is affected by communication range of the sensor node, which is enlarged by cooperative MIMO compared with single node. We show that the cooperative MIMO using MI-assisted synchronization and pure acoustic-based synchronization both have a great improvement in maximum transmission range compared with a single node in Figure 5. Therefore, the underwater MI-assisted acoustic cooperative MIMO networks can leverage the gains of MIMO in a distributed fashion to enhance connectivity of UWSNs. Meanwhile, MI communication works at ultra-high frequency and the signal transmission delay can be neglected. Thus, the influence of frequency and time estimation errors on its SNR is almost 0, and the maximum transmission range is almost the same as with perfect synchronization. However, in a pure acoustic-based synchronization system, due to the existence of error, the maximum transmission range is obviously less than the perfect synchronization.

**Figure 6:**
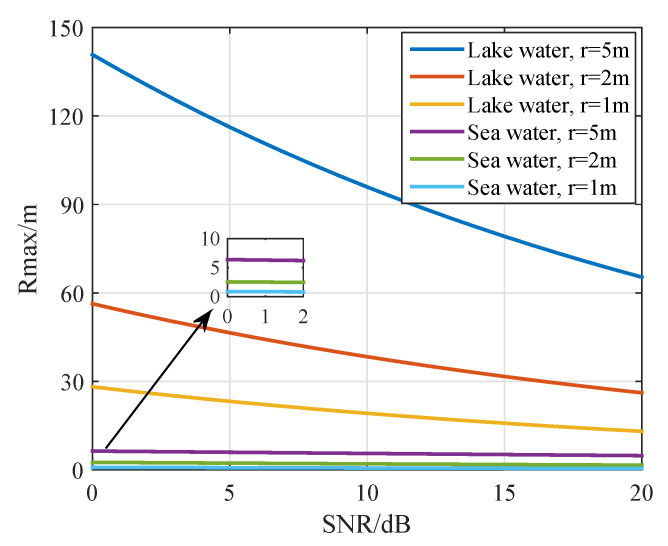
The intra-cluster maximum transmission range.

To explore the transmission range versus the variety of coil radius and SNR threshold, a simulation experiment was conducted, and the results are indicated in Figure 6. As shown in Figure 6, the range of the cluster can be extended by increasing the coil radius. Compared with underwater acoustic communication, MI communication has a smaller transmission range, so it is suitable for short distance inter-cluster communication. At the same time, it is worth noting that there is great difference in the transmission range of the MI communication between sea water and lake water. Highly conductive sea water induces significant Eddy current that incurs very high path loss, which results in the inevitable decrease of the transmission range. However, the transmission range of underwater MI communications can be increased by using the optimal operating frequency and larger coil antennas.

### 6.2. The Directly Connected Probability of the jth Cluster Consisting of k Nodes $P_{C,k,j}$

**Figure 7:**
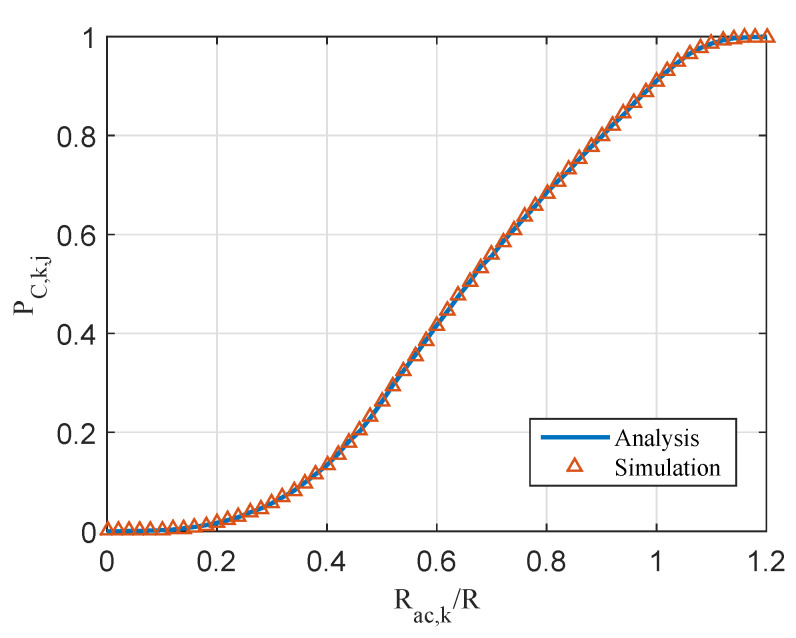
The directly connected probability of the *j*th cluster with consisting of *k* sensors $P_{C,k,j}$.

To get the directly connected probability of the *j*th cluster with consisting of *k* nodes, i.e., $P_{C,k,j}$, it is necessary to calculate the probability that the actual distance $R_{j}$ between the surface BS and the *j*th cluster is no more than the maximum transmission range $R_{ac,k}^{}$, i.e., $P_{C,k,j}=P\left(R_{j}\leq R_{ac,k}^{}\right)$. To obtain the statistical performance, 10,000 Monte Carlo simulations were performed. In a certain simulation, an underwater MI-assisted acoustic cooperative MIMO network was generated. If $R_{j}\leq R_{ac,k}$, the result was marked as 1; if $R_{j}>R_{ac,k}^{}$, the result was marked as 0. Finally, the probability $P_{C,k,j}$ was obtained by the 10,000 simulation results. As shown in Figure 7, the simulation results verify the correctness of the theoretical analysis in Equation (39).

### 6.3. Directly Connected Probability of the jth Cluster with Different SNR Threshold

**Figure 8:**
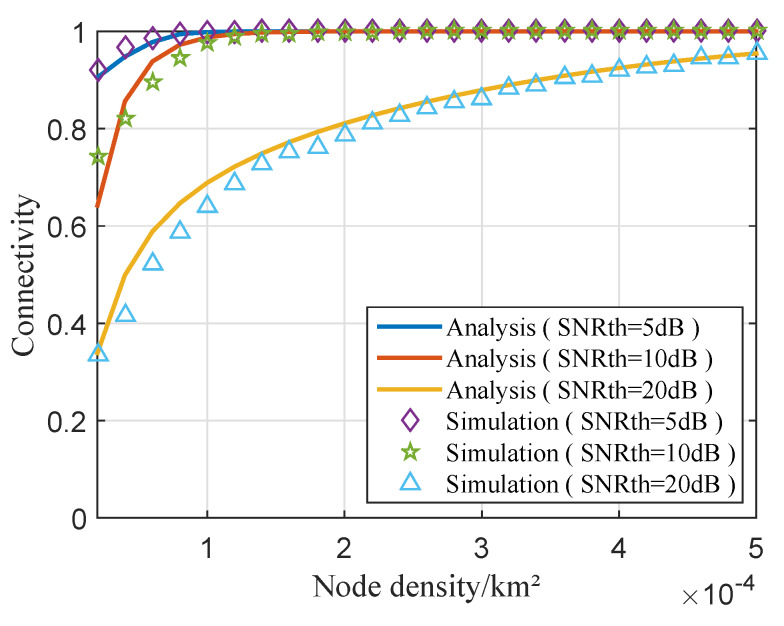
The directly connected probability of the *j*th cluster with different SNR thresholds.

The directly connected probability of the *j*th cluster with different SNR thresholds is investigated in Figure 8. To evaluate the performances of the proposed approach, 5000 Monte Carlo simulations were carried out in each case. Figure 8 shows that the higher SNR threshold leads to the smaller connectivity probability under the same node density. As $SNR_{th}$ increases from 5 to 20 dB, the transmission range of sensor gets shorter; as a result, fewer sensors participate in the cooperative MIMO, which means the smaller transmission range. Therefore, the probability of connectivity is lower. Note that, when node density is low, there is notable gap between analysis and simulation results. That is because we can gat that the transmission range of a cluster has a great influence on the theoretical analysis of its connectivity from Equation (39). While the cluster communicates with the BS in the form of cooperative MIMO, the number of nodes participating in the cooperative MIMO determines its transmission range. When the connectivity of the *j*th cluster is theoretically analyzed, the number of nodes may be non-integer, which is calculated by $\rho_{n}\pi R_{MI}^{2}$. However, the number of nodes in the cluster is determined in simulation. When the node density is low, there are few nodes participating in cooperative MIMO, and the non-integer number of nodes causes notable gap between analysis and simulation results, as shown in Figure 8.

### 6.4. Theoretical Bounds of the jth Cluster

**Figure 9:**
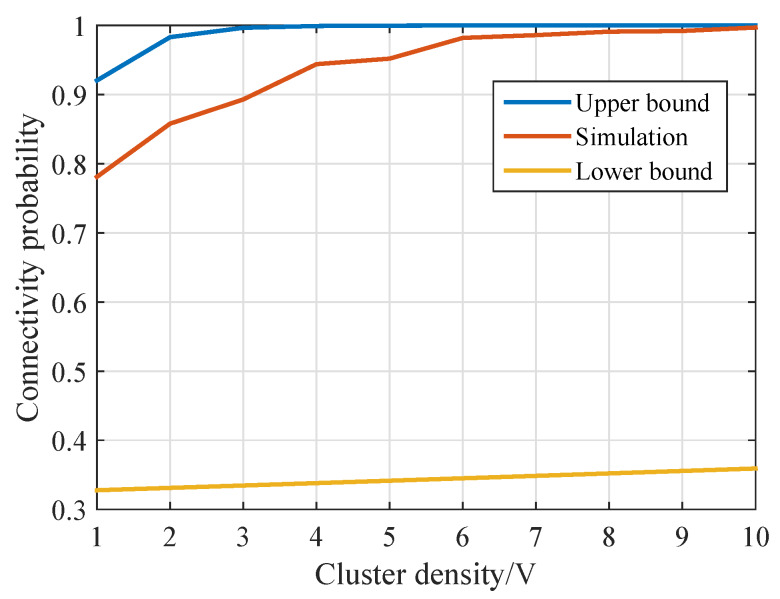
The theoretical bounds of the *j*th cluster with $SNR_{th}=20$ dB.

In Figure 9, the theoretical upper and lower bounds of underwater MI-assisted acoustic cooperative MIMO networks are compared with the simulation results with a variety of sensor densities. Each simulated connectivity probability was calculated based on 500 simulation iterations. The SNR threshold of the received signal was set to 20 dB. The connectivity probability increases as the cluster density increases. This result is consistent with the connectivity analysis and provide the guideline for the deployment of the networks according to the practical requirement.

### 6.5. Directly Connected Probability of the jth Cluster

**Figure 10:**
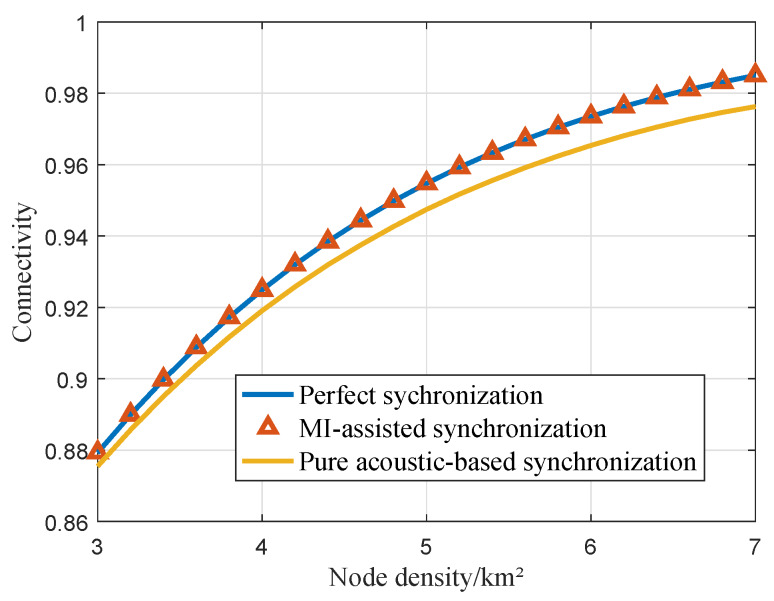
The directly connected probability of the *j*th cluster with $SNR_{th}=20$ dB.

The *j*th cluster’s directly connected probabilities in the cases of perfect synchronization, pure acoustic-based synchronization, and MI-assisted synchronization were investigated under the same simulation conditions, and the results are indicated in Figure 10. The connectivity probabilities increase as the node density increases. As the node density in the cluster becomes higher, it demonstrates that more sensors within a cluster participate in the cooperative MIMO, resulting in the long-range transmission. Considering the frequency and time estimation errors in the synchronization process, the maximum transmission range of the cluster is smaller than that of the perfect synchronization. Therefore, for the cluster with the same sensor density, the connectivity probability becomes smaller.

## 7. Conclusions

Recently, underwater MI-assisted acoustic cooperative MIMO networks have attracted much attention as they significantly improve the effective cover space and network throughput. However, the complex channel characteristics and the heterogeneous architecture make the connectivity more complex than that of traditional UWSNs. In this paper, we investigate the connectivity of the networks considering the influences of channel characteristics, system parameters, and synchronization error. To provide the theoretical guide for the design of underwater MI-assisted acoustic cooperative MIMO networks, the upper and lower bounds are both derived. Finally, Monte Carlo simulations were performed to address the correctness of the proposed mathematical models.
